# Robust optimization for casualty scheduling considering injury deterioration and point-edge mixed failures in early stage of post-earthquake relief

**DOI:** 10.3389/fpubh.2023.995829

**Published:** 2023-02-20

**Authors:** Yufeng Zhou, Ying Gong, Xiaoqin Hu

**Affiliations:** Research Center for Economy of Upper Reaches of the Yangtze River, Chongqing Technology and Business University, Chongqing, China

**Keywords:** emergency logistics, the injury worsened, facility disruption, robust optimization, particle swarm optimization

## Abstract

**Objective:**

Scientifically organizing emergency rescue activities to reduce mortality in the early stage of earthquakes.

**Methods:**

A robust casualty scheduling problem to reduce the total expected death probability of the casualties is studied by considering scenarios of disrupted medical points and routes. The problem is described as a 0-1 mixed integer nonlinear programming model. An improved particle swarm optimization (PSO) algorithm is introduced to solve the model. A case study of the Lushan earthquake in China is conducted to verify the feasibility and effectiveness of the model and algorithm.

**Results:**

The results show that the proposed PSO algorithm is superior to the compared genetic algorithm, immune optimization algorithm, and differential evolution algorithm. The optimization results are still robust and reliable even if some medical points fail and routes are disrupted in affected areas when considering point-edge mixed failure scenarios.

**Conclusion:**

Decision makers can balance casualty treatment and system reliability based on the degree of risk preference considering the uncertainty of casualties, to achieve the optimal casualty scheduling effect.

## 1. Introduction

Earthquakes often occur randomly without warning and bring devasting damages to affected areas. Earthquakes not only cause serious economic losses but also lead to many people's injuries or even death (1, 2). Statistically, the 1976 Tangshan earthquake caused 242,769 deaths and 435,556 injuries. The 2008 Wenchuan earthquake killed 69,227 people, injured 374,643 people, and left 17,923 people missing. After a large-scale destructive earthquake, the number of casualties increases rapidly, the injury states are complicated, and the affected areas are wide. The scientific and effective treatment of the casualties is the primary task of the post-disaster relief work, and its efficiency is related to the success or failure of the post-disaster rescue. Especially the early stage of post-earthquake relief is the critical period for emergency rescue and disaster relief (3). How to organize rescue activities plays a crucial role during this golden rescue period, such as transporting and treating the casualties (4). Consequently, utilizing existing medical resources to build an efficient and reliable emergency logistics network to transport the casualties to medical points efficiently for treatment is an urgent problem to be resolved in the early post-earthquake period.

The problem of casualty transportation scheduling was usually described as an emergency facility location-allocation problem or a delivery vehicle routing problem (5–8). On this basis, some studies considered the type of casualties (9, 10) or the deterioration of the wounded (11) to further optimize the casualty scheduling. However, the above literature generally contained an implicit assumption that the facility was completely reliable without disruption. This assumption did not correspond to reality. Especially in the post-earthquake emergency logistics system, the facilities and routings are easy to fail due to the impact of earthquake disasters and their secondary disasters. The disruption factors were considered in some literature (12–14).

To sum up, researchers have carried out in-depth research on the optimization of emergency scheduling for casualty transportation after earthquake disasters, but there are still some problems that are required further study. Firstly, most of the existing research studied the problem of injury deterioration and casualty scheduling alone without considering the two problems simultaneously. Secondly, most of the current studies assumed that the road and facilities in the earthquake disaster logistics network were completely reliable without failures. Although some literature considered road disruption or facility failures, there are few works considering the mixed failure state of points (facility) and edges (route) in the emergency logistics network simultaneously. Thirdly, existing studies usually assumed that information such as the number of casualties was determined or described as fuzzy parameters. In reality, such a number cannot be estimated exactly. To fill up this gap, this paper considers the point-edge mixed failure scenario, combines the evolution of injury situation, adopts the robust optimization method to deal with the uncertain amounts of casualties, and further studies the optimization problem of casualty transport scheduling in the early stage of post-earthquake relief.

The main differences between this paper and previous studies are summarized in Table 1. More specifically, the contributions of this paper are as follows. (1) We propose a new casualty scheduling robust optimization problem for post-earthquake relief. As mentioned in the previous subsection, the proposed problem considers some new characteristics in the early stage of post-earthquake relief, such as point-edge mixed failure scenarios, injury deterioration, and multiple transportation modes. (2) A mixed integer non-linear programming (MINP) model is established to formulate the proposed problem. (3) According to the characteristics of the model, an improved integer-encoded particle swarm optimization (PSO) algorithm is designed in this paper. The improved PSO is compared with the genetic algorithm (GA), immune algorithm (IA), and differential evolution algorithm (DE).

**Table 1:** Summary of relevant research.

**Reference**	**Road disruption**	**Facility disruption**	**Parameter uncertainty**	**Casualty triage**	**Injury deterioration**	**Multi-transportation modes**	**Solution method**	**Modeling approach**
Bronfman et al. (2)	No	No	No	Yes	Yes	No	CPLEX	MILP
Mansoori et al. (15)	No	No	Robust	No	No	No	CPLEX	MINP
Sun et al. (16)	No	No	Robust	Yes	Yes	Yes	CPLEX	MILP
Chou et al. (17)	No	No	No	No	No	No	Heuristic	MINP
Caunhye et al. (18)	No	No	Stochastic	Yes	No	No	Heuristic	MINP
Ghasemi et al. (19)	No	No	Stochastic	Yes	No	No	Heuristic	MINP
Caglayan et al. (20)	No	No	No	Yes	No	No	CPLEX	MINP
Vahdani et al. (21)	No	No	Robust	Yes	No	Yes	GAMS	MILP
Zeng et al. (22)	No	No	No	Yes	No	No	Heuristic	MILP
Cheng et al. (23)	Yes	Yes	Stochastic	No	No	No	Heuristic	MINP
Sun et al. (24)	No	Yes	Robust	Yes	No	No	CPLEX	MILP
Desi-Nezhad et al. (25)	Yes	No	Stochastic	No	No	Yes	GAMS and CPLEX	MILP
Our study	Yes	Yes	Robust	Yes	Yes	Yes	Heuristic	MINP

MILP, Mixed integer linear programming.

## 2. Literature review

This section will review relevant literature in the following three categories.

### 2.1. Traditional casualty scheduling problems

The problem of casualty transportation scheduling is usually described as an emergency facility location-allocation problem or a delivery vehicle routing problem (5–8). Mansoori et al. (15) proposed a multi-objective humanitarian supply chain design problem that minimizes the total number of injured not transferred to hospitals. Fiedrich et al. (26) proposed a dynamic optimization model where the total number of fatalities during the initial search-and-rescue period after strong earthquakes is minimized. Andersson et al. (27) established a support tool for dynamic ambulance relocation and automatic ambulance dispatching. Xie et al. (28) formulated a lane-based evacuation network optimization problem that integrates lane reversal and crossing elimination strategies. A Lagrangian relaxation algorithm based on the principles of Tabu search is designed to solve the model. Mclay et al. (29) proposed an improved Markov decision model to optimize ambulance dispatch dynamically to maximize the number of critically injured patients. Knyazkov et al. (30) studied the present situation of emergency transport for patients with acute coronary heart disease in St. Petersburg. The optimization of ambulance routes was proposed according to the road network in the city, to improve the number of ambulances per capita and the speed of ambulance response in this work. Sung et al. (31) transformed the problem of treating the injured after a disaster into the dispatching problem of emergency ambulances with the goal of maximizing the expected survival rate and designed a column generation algorithm. Repoussis et al. (32) proposed a mixed integer programming formulation for the combined ambulance dispatching, patient-to-hospital assignment, and treatment ordering problem. The objectives are to minimize the overall response time and the total flow time required to treat all patients. Shavarani et al. (33) described how to properly allocate existing emergency vehicles to hospitals and effectively plan vehicle routes after a disaster in a densely populated area, to maximize patient survival. Sun et al. (16) proposed an emergency model of the location-transportation-allocation problem. The objective is to minimize the total cost and the sum of injury severity scores. The integrated research on the problem of casualty scheduling and emergency facility location is studied in some literature (34, 35). Sheu et al. (36) proposed a method for designing a seamless centralized emergency supply network. A three-stage multi-objective (travel distance minimization, operational cost minimization, and psychological cost minimization) mixed-integer linear programming model is built to describe the problem. Hu et al. (37) proposed a mixed integer programming model that considers the uncertainty of the number of injured people and integrates the decision of locating shelters and transferring injured people. Chou et al. (17) proposed a patient transportation and assignment model considering the routing of ambulances and operational conditions of hospitals.

### 2.2. Casualty scheduling problems considering wounded types

A large number of wounded personnel emerged in the early stage after an earthquake, thus it is important to dispatch the wounded personnel considering the wounded type (9, 10). Caunhye et al. (18) constructed a three-stage stochastic programming model to locate alternative care facilities and allocate casualties. The model integrates casualty triage and the movement of self-evacuees. Ghasemi et al. (19) proposed a stochastic multi-objective mixed-integer mathematical programming for logistic distribution and evacuation planning during an earthquake. Na et al. (38) classified the wounded according to the results of the field diagnosis of the wounded, combined with the medical resources required by different wounded, assigned rescue vehicles to the wounded, and established a mixed integer linear programming model with cost minimization as the objective function. Talarico et al. (39) classified the injured into two categories: those that can be treated at local sites and those that must be treated in hospitals. They established an optimization model of ambulance routing decisions, aiming at minimizing the weighted waiting time of the injured, and solved the model with a large-scale neighborhood search algorithm. Rezapour et al. (40) divided the injured in each disaster area into yellow and red grades and studied how to reasonably allocate search and rescue personnel and medical personnel to the disaster area. More studies are available in literature (41, 42). The condition of the wounded deteriorates over time in reality. Wilson et al. (43) proposed a Markov chain model for the injury state transfer of casualties. Jin et al. (11) proposed an optimization model for patient delivery and medical resource allocation with capacity restrictions considering the severity of injuries. Liu et al. (44) set up a double objective optimization model for temporary medical service point location and the optimal medical service allocation decisions considering the deterioration of injury. The goals are to maximize the expected number of survival and minimize the total operation cost.

### 2.3. Casualty scheduling problems considering disruptions

The facilities (points) and the roads (edges) of an emergency logistics network might be disrupted after a large-scale earthquake. The logistics network design problem considering facility failures is studied in some literature. Bayram et al. (45) studied the shelter location and evacuation route assignment problem considering the disruption/degradation of the evacuation road network structure. Cheng et al. (23) adopted a two-stage robust optimization framework to study the robust fixed cost location problem in the case of uncertain demand and facility disruption, and developed a column constraint generation algorithm to accurately solve the model. Zhou et al. (46) constructed a location-allocation model for emergency facilities suitable for the initial stage of post-earthquake rescue, considering facility disruption and multiple types of fuzzy demand. Mohammadi et al. (47) studied a multi-objective reliable optimization model to organize a humanitarian relief chain. They made a broad range of decisions, including reliable facility location-allocation, fair distribution of relief items, assignment of victims, and routing of trucks. Sun et al. (24) proposed a scenario-based robust dual-objective optimization model to study the location of medical facilities, casualty transport, and relief material distribution under the temporary medical point failure scenarios. There are also some studies on the design of emergency logistics networks considering edge (road network) failures. Sabouhi et al. (48) proposed a comprehensive stochastic programming model for the distribution of relief materials in disaster areas, taking the demand and disrupted roads as uncertain parameters. Gong et al. (49) studied the decision optimization of patient scheduling in the early stage of post-earthquake rescue, considering the factors such as the deterioration of the injured and road disruption. Desi-Nezhad et al. (25) developed a two-stage stochastic programming model to transport injured people with consideration of multiple disruptions at transportation links and facilities.

## 3. Description of injury deterioration

The injury deterioration can be described by a Markov chain proposed by Wilson et al. (43). The injury state of the wounded evolves toward the direction of gradual aggravation or continues to maintain the original state, with a certain probability in the process of waiting for rescue. And death is the end of the injury evolution in process. If the injury at the current stage is severe, the injury may be transferred to the death state or remain severe if effective treatment is not available at the next stage. If the injury is minor at the current stage, the injury may be transferred to severe or remain minor if no rescue is available at the next stage. It can be seen from the evolution of the injury state that the state transfer is stochastic, and the state of the wounded at the next stage only depends on the current state independent of the historical state, which is consistent with the no aftereffect of the Markov process. Therefore, the evolution of the injury has a Markov character.

**Figure 1:**
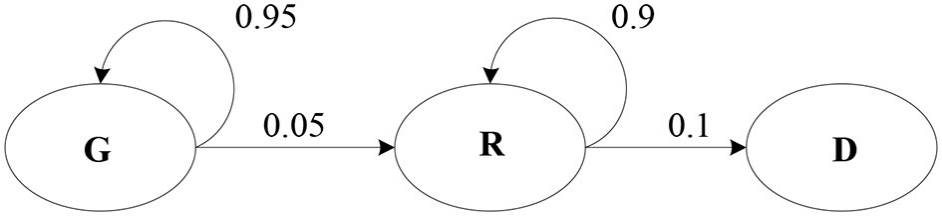
Random evolution process of the injury state.

In this paper, the evolution process of injury is divided into three finite-state Markov processes: minor injury (Green, G), severe injury (Red, R), and death (D). The process is repeated until the casualty dies or is saved. Let the initial time *t* = 0. All rescue teams are ready at the initial time. The injury level of the casualty is randomly and unidirectionally changed once per minute without any medical treatment, as shown in Figure 1.

It is assumed that there is a linear relationship between the severity of injury and the time before the wounded is treated. According to the random transfer probability between the injury states, the possible death probability of the casualty *j* whose initial injury state is *R* is expressed as formula (1).


(1) $$P_{r}^{D}(T_{ij}^{r},L_{r})=p_{rd}T_{ij}^{r}$$

In expression (1), $T_{ij}^{r}$indicates the time required to transport a severe casualty from the affected point *i* to the medical point *j*. *L*_*r*_ represents the injury state of the casualty in the initial time is *r*. *p*_*rd*_ indicates the probability that a casualty's injury state changes from severe to death and let *p*_*rd*_ = 0.1 in this article referring to literature (43).

If $p_{rd}T_{ij}^{r}>1$, the wounded may die in a waiting process. The death probability of the severe casualty while waiting for rescue can be expressed by Equation (2).


(2) $$P_{r}^{D}(T_{ij}^{r},\;L_{r})=\min\left(1,\;p_{rd}T_{ij}^{r}\right)$$

The death probability of the minor casualty can be written by the transition probability equation. That is, the probability of changing from a minor injury to a severe injury is calculated first, and then the probability of deteriorating from the severe injury to death is calculated. The time *T* that the wounded fully evolves from *G* to *R* should be first judged when calculating the death probability. According to Figure 1, the transition probability from state *G* to *R* is 0.05. Let 0.05^*^T = 1, then, *T* = 20 when *G* evolves into *R*. When the ambulance arrival time is <20 units, the wounded does not evolve to death, and the death probability is 0. When the arrival time of the ambulance is >20 units, the death probability in this state is $P_{g}^{D}\left(T_{ij}^{g},\;L_{g}\right)=\min\left(1,\;p_{rd}\left(T_{ij}^{\text{g}}-T\right)\right)$. So, the probability function of death in a minor state is a time-segment function, see expression (3).


(3) $$P_{\text{g}}^{D}(T_{ij}^{g},L_{g})=\left\{ \begin{array}{l} 0,\;0<T_{ij}^{g}\leq T\\ \min\left(1,p_{rd}(T_{ij}^{g}-T)\right),\;T_{ij}^{g}>T \end{array}\right.$$

Therefore, the death probability functional of a casualty can be expressed as (4).


(4) $$P_{w}^{D}(T_{ij}^{w},L_{w})=\left\{ \begin{array}{l} \min\left(1,\;0.1T_{ij}^{w}\right),\;w=r;\\ 0,\;w=g,0<T_{ij}^{w}\leq 20;\\ \min\left(1,0.1(T_{ij}^{w}-20)\right),\;w=g,T_{ij}^{w}>20; \end{array}\right.$$

## 4. Model

### 4.1. Problem description

**Figure 2:**
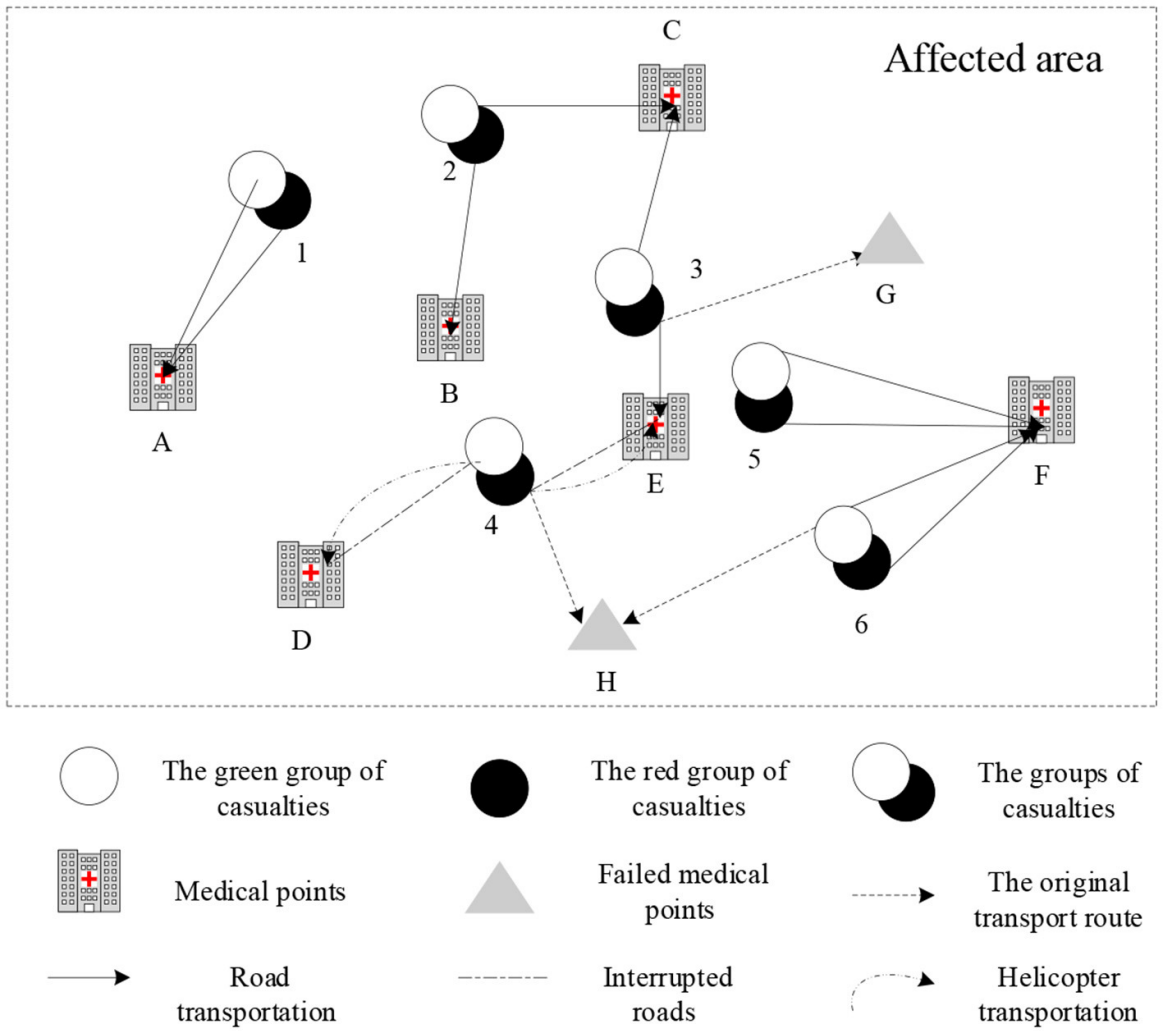
Transport network diagram in the early stage of post-earthquake relief.

The impact of aftershocks, mudslides, and other secondary disasters, not only leads to medical points failure but also causes road damage or interruption, in the early stage of post-earthquake relief. There is a mixed failure of facility nodes and routes in the emergency logistics network. The transport network diagram with the point-edge mixed failures in the early stage of post-earthquake relief is shown in Figure 2. As can be seen from Figure 2, based on the failures of facility points *H* and *G*, the secondary disaster also leads to the interruption of roads between affected point 4 and medical point *D*, and between affected point 4 and medical point *E*. At this time, ambulances cannot normally pass on the two roads, so it is necessary to use helicopters to transport the wounded to ensure timely treatment, reduce the death rate of the wounded, and improve the efficiency of emergency rescue.

The purpose of emergency rescue is to treat as many casualties as possible in the shortest time and reduce the death rate of the wounded. Therefore, the goal of the model is to minimize the expected death probability of casualties.

### 4.2. Assumptions

Model assumptions are as follows. First, the location of affected points and medical points is known, and the resources available at the medical point are limited. Second, each medical point can serve multiple casualty groups at the same time, and each casualty group can be assigned just one medical point. Third, the casualty groups have been classified in advance according to the trauma index of the injured. The severe injuries should be treated first according to the medical resources and ambulance and helicopter transport conditions. Four, each ambulance can only transport two casualties at a time, and each helicopter can transport four casualties at a time.

### 4.3. Notations

The parameters are as follows.

I: Set of affected points, *i* ∈ *I*;
J: Set of medical points, *j* ∈ *J*;
S: Set of Scenarios, *s* ∈ *S*;
R:The severe state of casualties (marked as red);
G:The minor injury state of casualties (marked as green);
W:Set of injury states,;
$P_{w}^{D}$:The death probability of casualties;
H: Set of ambulances, *h* ∈ *H*;
N: Set of rescue helicopters, *n* ∈ *N*;
M: A large positive integer number;

*d*_*ij*_: Transportation distance from affected point *i* to medical point *j*;

ε_*ij*_: Degree of route damage between affected point *i* and medical point *j*; if ε_*ij*_ > 0.5, the route is disrupted, and the ambulances cannot pass. Then, the helicopter transportation should be considered. With the support of modern remote sensing technology and communication technology, the damage degree of route in the early stage after earthquake can be sensed in real time without constant trial and error.

$d_{ij}^{\prime }$: Generalized transportation distance from affected point *i* to medical point *j*. The more damaged the route, the slower the vehicle speed, and the longer the transport time. That is, the longer the $d_{ij}^{\prime }$. $d_{ij}^{\prime }$ can be obtained according to the method mentioned in literature (50), as $d_{ij}^{{}^{\prime }}=\left\{ \begin{array}{l} d_{ij}(1+\varepsilon_{ij})\;\varepsilon_{ij}\leq 0.5\\ \;M\;\varepsilon_{ij}>0.5 \end{array}\right.$.

*t*_*ij*_: The time taken to transport the wounded from affected point *i* to medical point *j*;

T: The horizon of scheduling time,∀*t* ∈ *T*;

$\text{Δ}T_{ij}^{w}$: The waiting time for ambulances or helicopters rescue for a casualty with injury state *w*;

*v*_*h*_: The speed of ambulance *h*;

*v*_*n*_: The speed of helicopter *n*;

*Q*_*j*_: The number of available resources at medical point *j*;

*Q*_*h*_: The number of available ambulance *h* at medical points;

*Q*_*n*_: The number of available helicopter *n* at medical points;

*q*_*h*_: The number of casualties can be transported by ambulance *h* at one time;

*q*_*n*_: The number of casualties can be transported by helicopter *n* at one time;

*C*_*w*_: Resources needed to treat a casualty with injury state *w*, *W* = {*w*|*w* = *r, g*};

a: Number of times an ambulance or a helicopter arrived at an affected point;

*f*_*iw*_: The number of casualties with injury state *w* in affected point *i*;

β_*js*_: β_*js*_ = 0 represents the medical point *j* fail in scenario *s*; otherwise β_*js*_ = 1.

The decision variables are as follows.

*k*_*ijw*_: The number of casualties with injury state *w* transported from affected point *i* to medical point *j*;

*X*_*ij*_: A binary variable. If medical point *j* allocated to affected point *i*, *X*_*ij*_ = 1; otherwise *X*_*ij*_ = 0;

*X*_*i′j*_: A binary variable. If medical point *j* reallocated to affected point *i*′ after the initial allocated medical point *j*′ failed, $X_{i^{\prime }j}=1$;otherwise $X_{i^{\prime }j}=0$;

$Y_{i^{\prime }jh}$: A binary variable. If ambulance *h* travels from affected point *i*′ to medical point *j* after the initial allocated medical point *j*′ failed, $Y_{i^{\prime }jh}=1$; otherwise $Y_{i^{\prime }jh}=0$;

$Y_{i^{\prime }jh}$: A binary variable. If ambulance *h* travels from affected point *i*′ to medical point *j* after the initial allocated medical point *j*′ failed, *Y*_*ijh*_ = 1; otherwise *Y*_*ijh*_ = 0;

*Z*_*ijn*_: A binary variable. If helicopter *n* travels from affected point *i* to medical point *j*, *Z*_*ijn*_ = 1; otherwise *Z*_*ijn*_ = 0;

$Z_{i^{\prime }jn}$: A binary variable. If helicopter *n* travels from affected point *i*′ to medical point *j* after the initial allocated medical point *j*′ failed, $Z_{i^{\prime }jn}\text{=}1$; otherwise $Z_{i^{\prime }jn}=0$.

### 4.4. Mathematical formulation

The mathematical model can be formulated as follows.


(5) $$Z=\min\sum_{w\in W}f_{iw}P_{w}^{D},\;\forall i\in I,j\in J$$

(6) $$\sum_{w\in W}P_{w}^{D}=\;\left\{ \begin{array}{l} \sum_{w\in W}\min\left(1,\;0.1\Delta T_{ij}^{w}\right),\;w=r\;\\ 0,\;w=g,0<\Delta T_{ij}^{w}\leq 20;\;\forall i\in I,j\in J\\ \sum_{w\in W}\min\left(1,\;0.1(\Delta T_{ij}^{w}-20)\right),\;w=g,\Delta T_{ij}^{w}>20 \end{array}\right.$$

(7) $$\sum Q_{j}\geq \sum_{h\in H}\sum_{w\in W}C_{w}k_{ijw},\;\forall i\in I,j\in J,s\in S$$

(8) $$Q_{j}^{0}=Q_{j},\;\forall j\in J$$

(9) $$Q_{j}^{t}=Q_{j}^{t-1}-{\sum_{w\in W}C_{w}k_{ijw}}^{t-t_{ij}-1},\;\forall i\in I,\;j\in J,\;t\in T$$

(10) $$\sum_{h\in H}aQ_{h}q_{h}\text{​}\geq \text{​}\sum_{h\in H}\text{​}\sum_{w\in W}\text{​}k_{ijw}Y_{ijh}\text{​}+\text{​}\sum_{h\in H}\sum_{w\in W}k_{i\prime jw}Y_{i\prime jh},\;\forall i,i\prime \in I,j\in J$$

(11) $$\sum_{n\in N}\text{​}aQ_{n}q_{n}\text{​}\geq \text{​​}\sum_{n\in N}\text{​}\sum_{w\in W}k_{ijw}Y_{ijn}\text{​}+\text{​}\sum_{n\in N}\sum_{w\in W}k_{i\prime jw}Y_{i\prime jn},\;\forall i,i\prime \in I,j\in J$$

(12) $$\text{Δ}T_{ij}^{r}=\left\{ \begin{array}{l} (2a-1)t_{ij},Q_{j}^{t}-k_{ijr}C_{r}-k_{ijg}C_{g}\geq C_{r},\;\forall i\in I,j\in J,t\in T\\ \;M,\;Q_{j}^{t}-k_{ijr}C_{r}-k_{ijg}C_{g}\geq C_{r} \end{array}\right.$$

(13) $$\text{Δ}T_{ij}^{r}=\left\{ \begin{array}{l} (2a-1)t_{ij},Q_{j}^{t}-k_{ijr}C_{r}-k_{ijg}C_{g}\geq C_{g},\;\forall i\in I,j\in J,t\in T\\ \;M,\;Q_{j}^{t}-k_{ijr}C_{r}-k_{ijg}C_{g}\geq C_{g} \end{array}\right.$$

(14) $$t_{ij}=\left\{ \begin{array}{l} d_{ij}(1+\varepsilon_{ij})\;\varepsilon_{ij}\leq 0.5\;\forall i\in I,j\in J\\ \;M\;\varepsilon_{ij}\leq 0.5 \end{array}\right.$$

(15) $$t_{ij}=\{\begin{array}{l} \frac{{d^{\text{′}}}_{ij}}{v_{h}}X_{ij}\beta_{js}+\frac{{d^{\text{′}}}_{i\text{′}j}}{v_{h}}X_{i^{\text{′}}j}\text{ ​​}\varepsilon_{ij}\text{​​ }\leq 0.5\\ \frac{d_{ij}}{v_{n}}X_{ij}\beta_{js}+\frac{d_{i\text{′}j}}{v_{n}}X_{i^{\text{′}}j}\;\varepsilon_{ij}>0.5\; \end{array},\;\forall i,i^{\text{′}}\in I,j\in J,s\in S$$

(16) $$X_{i^{\prime }j}=\left\{ \begin{array}{l} 1,\left\{j|\cap \left(\underset{j\in J^{\prime }}{\min}t_{i^{\prime }j},\beta_{js}=1,\beta_{j^{\prime }s}=0\right)\right\},\\ 0,\;otherwise \end{array}\right.\forall i,i^{\prime }\in I,j\in J,s\in S$$

(17) $$X_{ij},X_{i^{\prime }j},Y_{ijh},Y_{i^{\prime }jh},Z_{ijn},Z_{i^{\prime }jn}\in \left\{0,1\right\},\;\forall i,i^{\prime }\in I,j\in J$$

The objective function (5) is to minimize the total expected death probability of casualties. Constraint (6) denotes the death probability of casualties with different injury states. Constraint (7) is the resource limitation of medical points. Constraint (8) denotes the initial resources of medical points. Constraint (9) denotes the restriction of available resources at medical points at time *t*. Constraints (10) and (11) denote the restrictions on the number of available ambulances and helicopters from affected points to medical points respectively. Constraints (12) and (13) denote the waiting time for casualties with injury state *g* and *r*, respectively. Constraint (14) denotes the transportation distances from affected points to medical points. Constraint (15) denotes the time taken to transport casualties from affected points to medical points. Constraint (16) denotes each affected point will be served by the nearest open backup medical point if its initial allocated medical point has failed. Constraint (17) is a 0–1 variables restriction.

### 4.5. A robust optimization model

As the destructive earthquake occurred suddenly, and aftershocks may result in injuries in the initial stage after an earthquake, the number of casualties in affected points cannot be accurately estimated. Therefore, robust optimization was used to deal with the number of casualties to reduce the risk caused by uncertainty.

The parameter Γ_*iw*_ and corresponding variables are introduced to process the objective function and constraint Referring (51). Objective function equation (5) has uncertain variables of *f*_*iw*_. Define the value range of ${\tilde{f}}_{iw}$ as $\left[f_{iw}-{\hat{f}}_{iw},f_{iw}+{\hat{f}}_{iw}\right]$. *f*_*iw*_ is the nominal value of uncertain number of casualties. ${\hat{f}}_{iw}$is the maximum deviation of the uncertain number of casualties. We introduce variable *f* to transform the objective function (5) referring to (52). The objective function (5) is equivalent to (18)–(19).


(18) min f

(19) $$\sum_{w\in W}{\tilde{f}}_{iw}P_{w}^{D}\leq f$$

A protection function for the number of casualties is introduced here as equation (20). Γ_*iw*_ is the control coefficient of the number of casualties.

A protection function for the number of casualties is introduced here as equation (20). Γ_*iw*_ is the control coefficient of the number of casualties. Γ_*iw*_ ∈ [0, |*J*_*iw*_|]. Where *J*_*iw*_ is the number of Γ_*iw*_. The objective of this robust method is that the number of casualties varies within their interval at most ⌊Γ_*iw*_⌋ affected points. Γ_*iw*_ is the balance between robustness and optimality. The bigger the Γ_*iw*_, the more conservative the mode. The protection function for the number of casualties is introduced as with φ(*X*, Γ_*iw*_) consideration of the uncertainty of the number of casualties. The objective function (5) can be transformed into formula (20).


(20) $$\begin{array}{l} \varphi (X,\Gamma_{iw})=\underset{\left\{S_{iw}ᑌ\left\{t_{iw}\right\}|S_{iw}\subseteq J_{iw},\left|S_{iw}\right|=\left\lfloor \Gamma_{iw}\right\rfloor ,t_{iw}\in J_{iw}\backslash S_{iw}\right\}}{\max}\\ \left\{\sum_{i,w\in S_{iw}}{\hat{f}}_{iw}P_{w}^{D}+\left(\Gamma_{iw}-\left\lfloor \Gamma_{iw}\right\rfloor \right){\hat{f}}_{t_{iw}}P_{w}^{D}\right\} \end{array}$$

Where, *S*_*iw*_ represents the group set of the maximum number of casualties deviating from the nominal value. When Γ_*iw*_ = 0, ∀*i, w*, the robust model is equivalent to the nominal model.

Formula (20) can be expressed as (21).


(21) $$\begin{array}{l} \sum_{w\in W}\{f_{iw}P_{w}^{D}+\underset{\left\{S_{iw}ᑌ\left\{t_{iw}\right\}|S_{iw}\subseteq J_{iw},\left|S_{iw}\right|=\left\lfloor \Gamma_{iw}\right\rfloor ,t_{iw}\in J_{iw}\backslash S_{iw}\right\}}{\max}\\ \left\{\sum_{i,w\in S_{iw}}{\hat{f}}_{iw}P_{w}^{D}+\left(\Gamma_{iw}-\left\lfloor \Gamma_{iw}\right\rfloor \right){\hat{f}}_{t_{iw}}P_{w}^{D}\right\}\}\leq f,\forall i\in I,j\in J \end{array}$$

If Γ_*iw*_, ∀*i, w* is a integer,Γ_*iw*_ = ⌊Γ_*iw*_⌋, ∀*i, w*, then


(22) $$\begin{array}{l} \sum_{w\in W}\{f_{iw}P_{w}^{D}+\underset{\left\{S_{iw}ᑌ\left\{t_{iw}\right\}|S_{iw}\subseteq J_{iw},\left|S_{iw}\right|=\left\lfloor \Gamma_{iw}\right\rfloor ,t_{iw}\in J_{iw}\backslash S_{iw}\right\}}{\max}\\ \left\{\sum_{i,w\in S_{iw}}{\hat{f}}_{iw}P_{w}^{D}\right\}\}\leq f,\forall i\in I,j\in J \end{array}$$

If Γ_*iw*_ = 0, ∀*i, w*,all the number of casualties are nominal. When Γ_*iw*_ = γ, the number of casualties in all injury states deviates from the nominal values, the model is equivalent to the Soyster model. As Γ_*iw*_ changes, the conservatism of the model also changes accordingly. The objective function (5) can be finally transformed into the following expressions based on the strong duality.


(23) $$\sum_{w\in W}f_{iw}P_{w}^{D}+Z_{iw}\Gamma_{iw}+\sum_{i\in J_{iw}}P_{iw}\leq f$$

(24) $$s.t.\left\{ \begin{array}{l} {\text{Z}}_{iw}+P_{iw}\geq {\hat{f}}_{iw}P_{w}^{D}\\ \;P_{iw}\geq 0,\\ \;{\text{Z}}_{iw}\geq 0,\\ \forall i,w\in S_{iw},\forall w\in W \end{array}\right.$$

Therefore, the robust optimization model considering the uncertain number of casualties can be given as follows.


(25) $$\begin{array}{l} \text{ min }f\\ s.t.(6)-(17). \end{array}$$

## 5. Algorithm

The built model is a 0–1 mixed integer non-linear programming model, which cannot be solved by exact algorithms such as branch and bound algorithm or operations research software such as CPLEX. An improved integer-coded PSO algorithm is designed to solve the model.

The algorithm steps are described as follows.

### 5.1. Population initialization

① Initialization of parameters: Set population size as *popsize*, maximum iterations as max*gen*, learning factors as *c*_1_ and *c*_2_, inertia weight as *w*.

**Figure 3:**
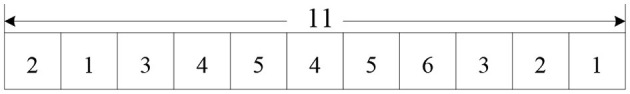
A diagram of particle coding.

② Particle encoding and decoding: Supposing there are *n* casualty groups and *k* medical points in the model, each particle has a code length of *n*. Each position of the particle is a positive integer randomly generated between 1 and k, and denotes the relation of assign between a casualty group and a medical point. Taking Figure 3 for example, there are 6 medical points in the affected area, which are required to provide rescue services to 11 casualty groups. The length of the particle is 11. Casualty group 2 and 11 are assigned to medical point 1, casualty group 1 and 10 are assigned to medical point 2, and so on.

③ Initialization of the best value of individuals and groups: The initial location and velocity of particles can be randomly generated. The fitness value of the current population can be calculated by the fitness function. The fitness function is the objective function (25). The individual position is the optimal position of the current individual *P*_*best*_. The minimum value of the current particle *P*_*best*_*value*_ is the initial optimal value of individuals that can be got by comparing the optimal values of all individuals. Then set the initial *P*_*best*_*value*_ as the best value of groups *g*_*best*_.

### 5.2. Updating speeds and locations

Population speed and location can be updated as expressions (26) and (27).


(26) $$v_{id}=w^{*}v_{id}+c_{1}r_{1}(p_{id}-x_{id})+c_{2}r_{2}(p_{gd}-x_{id})$$

(27) $$x_{id}=x_{id}+v_{id}$$

In which, *r*_1_,*r*_2_ are uniform random numbers within the range of [0,1].

**Figure 4:**
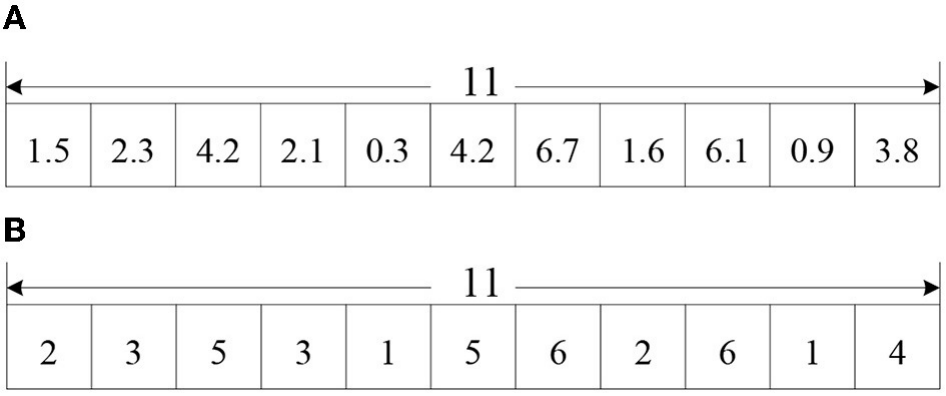
**(A)** Updated particle position. **(B)** Updated particle position round up to integers.

Traditional PSO is mainly used to solve the continuous optimization problem, while the model in this paper belongs to a discrete combinatorial problem. So, the updated particle positions and speeds need integer processing. Figure 4A is the result of an updating particle according to the particle update equations, and Figure 4B is the result of a particle rounds up to integers. Here, casualty group 5 and 10 are assigned to medical point 1, casualty group 1 and 8 are assigned to medical point 2, and so on.

### 5.3. Updating the best value of individuals

Comparing the current particle fitness value *fitness* with the individual historical optimal value *P*_*best*_*value*_, the smallest value is taken as the current individual optimal value *P*_*best*_*value*_, and its corresponding position is taken as the current individual optimal position *P*_*best*_.

### 5.4. Updating the best value of groups

Comparing the current individual optimal value *P*_*best*_*value*_ with the group historical optimal value *g*_*best*_*value*_, the smallest value is taken as the current group optimal value *g*_*best*_*value*_, and its corresponding position is taken as the current group global optimal position *g*_*best*_.

### 5.5. Termination of the algorithm

Determining whether the algorithm reaches the maximum iteration, if so, the algorithm terminates and outputs the optimal solution. Otherwise, go to step 4.2 and continue the iteration.

## 6. Computational experiments

### 6.1. Case description and data

The data of emergency medical rescue for Ya'an, in the Lushan earthquake, Sichuan province of China is used for numerical simulation. Taking town or township as units, Lushan County has jurisdiction over 5 towns and 4 townships. So, 9 affected points are set in this article. The nominal number of casualties in each affected point is shown in Supplementary Table S1. The number of treatment resources required by casualties with injury state *r* and *g* is 3 units and 2 units respectively.

Data shows that the Chinese government and military deployed 15 helicopters to transport and rescue casualties in the Lushan earthquake. Therefore, the number of available helicopters in medical points is set as 15. According to the basic Standard for Medical Institutions (Trial) issued by the Chinese Ministry of Health, there is one ambulance for every 50,000 people in the city. The number of ambulances in Ya'an city and Chengdu city is about 30 and 300, respectively. In this paper, it is assumed that 50% of ambulances in Ya'an city can be used normally, and due to the time crunch, only 50% of ambulances from nearby Chengdu city are used for the transport of casualties. So, the total number of available ambulances in this paper is set as 165. The other parameters to the model are set as following. *C*_*r*_ = 3 units, *C*_*g*_ = 2 units, *v*_*h*_ = 40 km/h, *v*_*n*_ = 120 km/h, *q*_*h*_ = 2 persons, *q*_*n*_ = 4 persons, *T* = 30 h. The number of medical resource limitations for each medical point is shown in Supplementary Table S2. The distance between medical points and affected points is shown in Supplementary Table S3.

Referring to literature (46), four failure scenarios are set in Supplementary Table S4. The failure scenarios are set as follows. No medical point failure in scenario 1, medical point 2 failure in scenario 2, medical point 4 failure in scenario 3, medical point 7 failure in scenario 4. 0 means the medical point failure in Supplementary Table S4. Supplementary Table S5 shows the degree of route damage ε_*ij*_.

### 6.2. Calculation results

**Figure 5:**
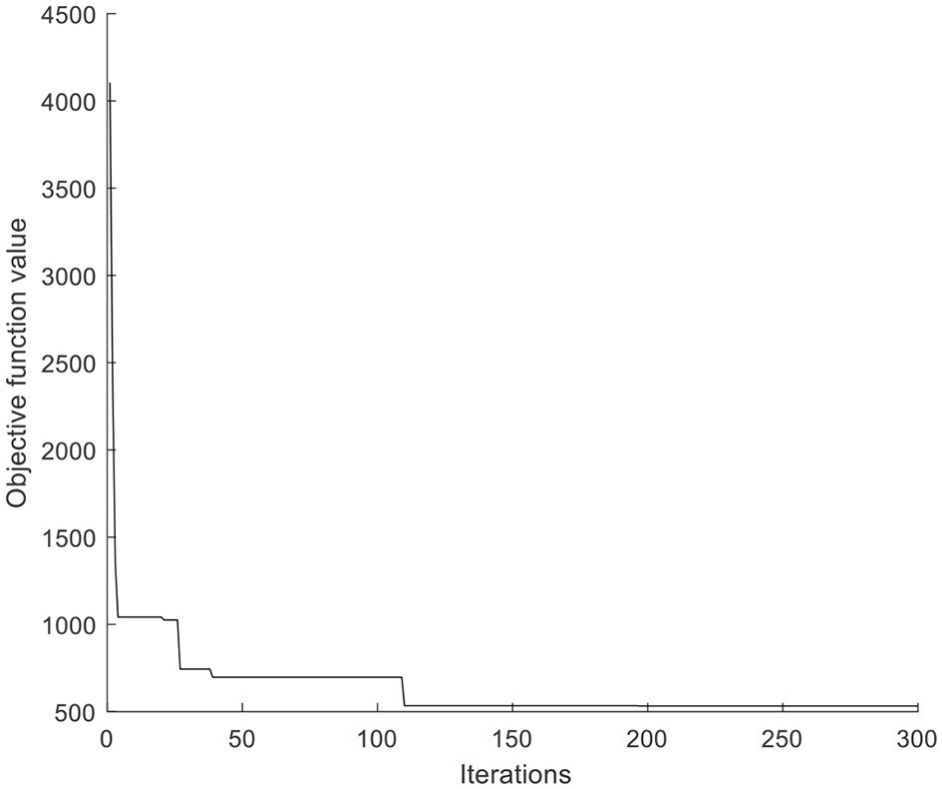
The convergence diagram of PSO.

The PSO parameters are set as follows. Population size *popsize* = 100, learning factors *c*_1_ = *c*_2_ = 2, inertia weight *w*= 1, the maximum iterations max*gen* = 300. MATLAB R2018b is used for programming, and it runs on a notebook computer with Intel(R) Core (TM) I5-5200U CPU and 12G memory. The optimal solution of the results of 10 operations is taken as the final solution. The convergence time of the algorithm is 10.171 s, and the objective function is 531.629. The convergence diagram of the algorithm is shown in Figure 5. Table 2 and Supplementary Table S6 are treatment information at medical points and affected points respectively.

**Table 2:** Treatment information at medical points.

**Medical points**	**Casualty group with injury state *r***	***k*_*jr*_**	**Casualty group with injury state *g***	***k*_*jg*_**	***k*_*jw*_**
1	R1, R2, R3	291	–	0	291
2	R6	178	G3	142	320
3	–	0	G2, G6	540	540
4	R4	116	G1	292	408
5	R7	42	G4, G5	324	366
6	R5, R8	126	G7, G8	222	348
7	R9	20	G9	40	60

### 6.3. Considering failures vs. without considering failures

To test the reliability of the scheme got by considering the point-edge mixed failures, a comparison was made between two schemes. Scheme 1 is the current optimization results got by considering the point-edge mixed failures. Scheme 2 is the results got without considering the failures. It is noted that the original objective function value in scheme 2 contains no factors of failures. The objective function value under each scenario in scheme 2 should be recalculated considering the facility failures and road disruption again (Table 3).

**Table 3:** Comparison of the two schemes.

**Schemes**	**Scenarios**	**Scenario 1**	**Scenario 2**	**Scenario 3**	**Scenario 4**
1	Considering the point-edge mixed failures	531.629	531.629	531.629	531.629
2	Without considering the failures	459.334	751.217	851.767	612.256
–	Gap	−13.60%	41.30%	60.22%	15.17%

The results show that when there is no failure (scenario 1), the casualty scheduling results under scheme 1 are slightly worse than those under scheme 2. However, when the failure occurs after the earthquake, the solution result considering the failures is better than that without considering the failures. As shown in scenario 3, the total expected death probability of casualties under scheme 2 was 60.22% higher than scheme 1. Therefore, considering the point-edge mixed failure in advance can make more casualties receive timely treatment and reduce the death rate. The reliability of the system can be improved by considering the failures in the design stage of the emergency rescue network.

### 6.4. Robust optimization vs. deterministic optimization

To verify the effectiveness of the robust optimization model, the running results of the robust optimization model and the deterministic model were compared in the same scenario. Four numerical examples are designed according to different control coefficients Γ_*w*_, and the maximum disturbance value of casualty number deviating from the nominal value is 20%, that is ${\hat{f}}_{iw}=0.2\times f_{iw}$. Table 4 shows the sensitivity analysis results of different robust control coefficients Γ_*w*_. In general, although the objective function value of the robust optimization model is higher than that of the deterministic model, the gap is not significant. It shows that the robust optimization model can reduce uncertain risk. From the results of sensitivity analysis of Γ_*w*_, the smaller the Γ_*w*_, the stronger the robustness of the model. With the increase of the uncertainty of casualty numbers, the objective function value increases. The gap between the robust optimization model and the deterministic model (when Γ_*w*_ is 0) is gradually increasing. The appropriate scheme should be chosen according to the uncertain situation in practical decision-making.

**Table 4:** Sensitivity analysis results of Γ_*w*_.

**Γ_*w*_**	**Objective function value**	**Gap**
0	561.553	0.00%
2	577.834	2.90%
4	595.820	6.10%
8	596.725	6.26%

### 6.5. Algorithm performance analysis

**Figure 6:**
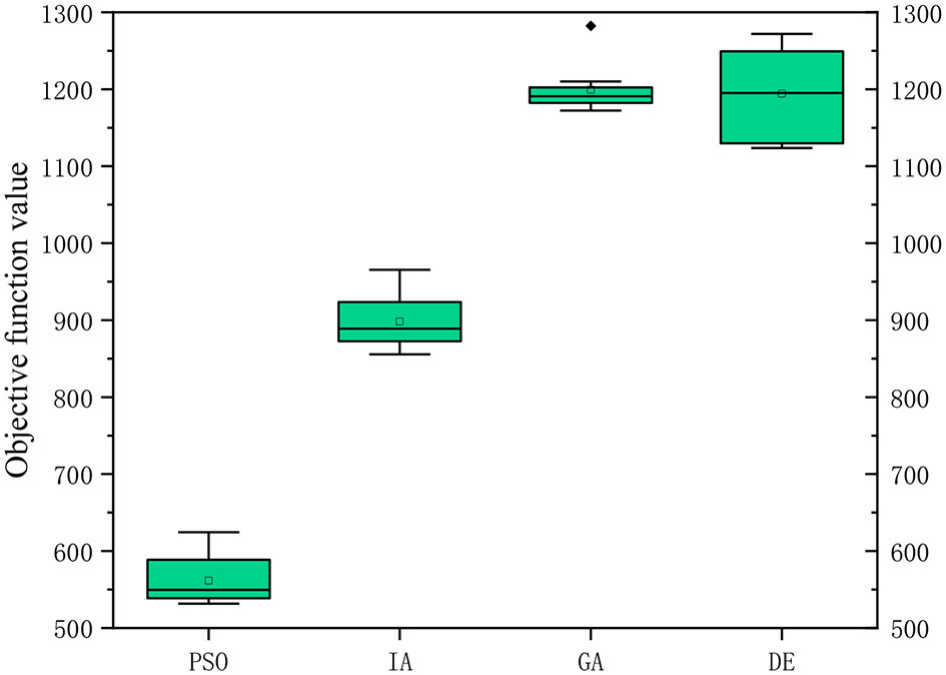
Computational accuracy comparison of the four algorithms.

To test the performance of PSO, the algorithm was compared with GA, IA and DE. Each algorithm was run 10 times. The results are shown in Table 5 that the PSO is significantly superior to other algorithms. The average objective function value calculated by PSO reduces by 53.2% compared with GA, 37.5% compared with IA, and 53.0% compared with DE. The computational accuracy of PSO is better than GA, IA and DE, as shown in Figure 6.

**Table 5:** Comparison of the results solved by different algorithms.

**Algorithm**	**Objective function value**				**CPU time (s)**

	**Min**	**Max**	**AVG**	**SD**	
PSO	531.629	624.441	561.553	30.905	10.171
IA	855.691	965.369	898.150	35.622	12.762
GA	1,172.350	1,282.256	1,193.264	31.381	9.178
DE	1,123.614	1,272.032	1,194.355	56.335	8.886

**Figure 7:**
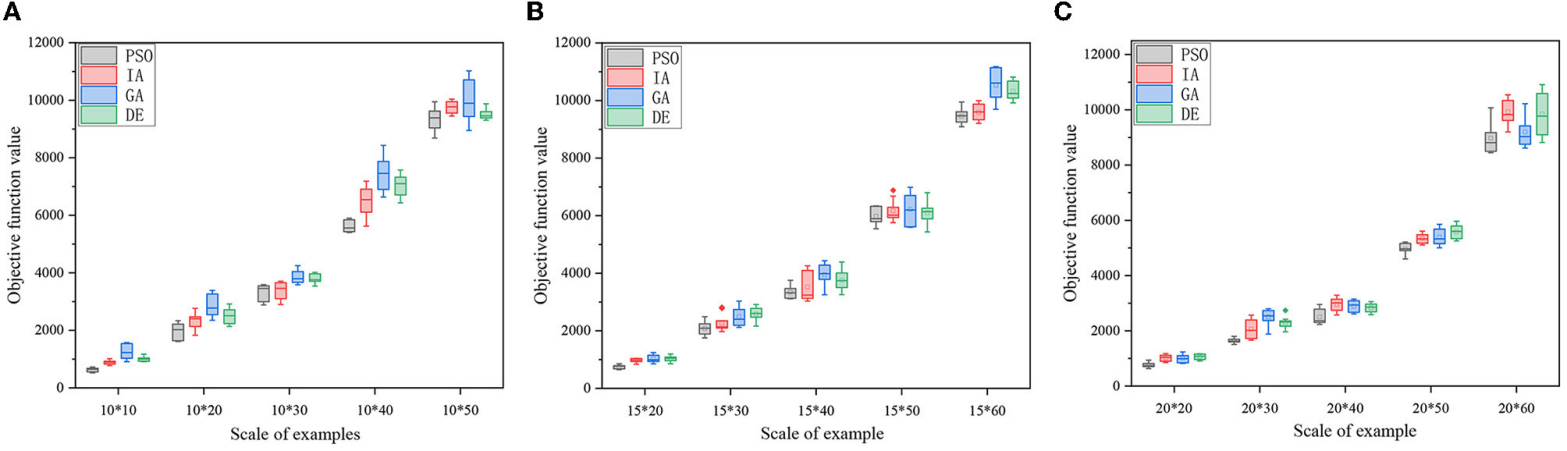
**(A)** Box diagram of group 1. **(B)** Box diagram of group 2. **(C)** Box diagram of group 3.

To further test the performance of the algorithm, three groups of examples are set for scale analysis. Each group contains five examples. The three groups have 10, 15 and 20 candidate medical points respectively. The calculation results are shown in Tables 6–8, and the box diagrams of the four algorithms for the three groups are shown in Figures 7A–C. The results show that PSO is better than GA, IA and DE obviously in terms of calculation accuracy. Among the 10 examples, only one example IA is better than PSO. The computational accuracy of PSO is better than GA, IA and DE for most examples. Therefore, the PSO designed in this paper can increase the optimization ability of the algorithm and has a good performance.

**Table 6:** Scale analysis of the group 1.

**Nodes**	**Algorithm**	**Objective function value**			**GAP**	**CPU time (s)**
		*Z* ^*^	*Z*−*MAX*	*Z*−*AVG*		
10^*^10	PSO	518.290	724.181	628.742	–	13.886
	IA	775.670	1,010.727	881.677	33.18%	15.268
	GA	909.409	1,573.435	1,265.659	43.01%	10.267
	DE	910.378	1,167.492	1,000.632	37.17%	10.818
10^*^20	PSO	1,610.292	2,331.576	1,961.454	–	26.414
	IA	1,825.655	2,764.848	2,331.196	11.80%	26.985
	GA	2,348.573	3,265.085	2,852.104	28.39%	24.359
	DE	2,133.259	2,917.516	2,480.780	20.93%	23.422
10^*^30	PSO	2,882.112	3,589.796	3,319.307	–	38.898
	IA	2,900.485	3,706.958	3,392.738	0.63%	40.256
	GA	3,580.927	4,248.524	3,859.806	19.51%	39.245
	DE	3,539.897	4,017.549	3,790.272	12.43%	38.429
10^*^40	PSO	5,403.128	5897.38	5,605.815	–	51.517
	IA	5,621.404	7,186.703	6,506.498	3.88%	53.541
	GA	6,636.561	8,432.381	7,456.442	18.59%	53.149
	DE	6,432.144	7,571.784	7,043.505	20.41%	52.498
10^*^50	PSO	8,684.691	9,947.969	9,320.860	–	64.694
	IA	9,452.603	10,039.604	9,750.315	8.12%	70.845
	GA	8,952.025	11,020.991	10,021.034	2.99%	65.754
	DE	9,308.26	9,877.368	9,501.766	1.90%	64.691

*Z*, Objective function value; Z^*^, the best objective function value; *Z*−*AVG*, the average value of *Z*; GAP, [Z^*^(algorithm) – Z^*^(PSO)]/Z^*^(algorithm)^*^100%.

**Table 7:** Scale analysis of the group 2.

**Nodes**	**Algorithm**	**Objective function value**			**GAP**	**CPU time (s)**
		*Z* ^*^	*Z*−*MAX*	*Z*−*AVG*		
15*20	PSO	650.165	856.959	753.769	–	34.923
	IA	839.106	1,044.125	972.196	22.52%	36.215
	GA	856.959	1,243.259	1,021.565	24.13%	35.512
	DE	856.497	1,191.873	1,029.144	26.76%	34.048
15^*^30	PSO	1,752.119	2,488.724	2,078.086	–	50.588
	IA	1,973.053	2,815.589	2,257.744	11.20%	54.154
	GA	2,118.788	3,032.033	2,487.702	17.31%	50.455
	DE	2,162.947	2,914.494	2,579.405	19.44%	49.081
15^*^40	PSO	3,108.708	3,754.086	3,346.931	–	65.382
	IA	3,032.033	4,257.05	3,524.326	−2.53%	71.452
	GA	3,251.877	4,433.367	3,949.915	4.40%	64.514
	DE	3,257.023	4,390.083	3,759.763	10.98%	64.017
15^*^50	PSO	5,543.338	6,340.27	5,981.089	–	78.216
	IA	5,758.349	6,878.194	6,165.836	3.73%	82.041
	GA	5,596.413	6,983.324	6,219.371	0.95%	81.463
	DE	5,436.843	6,799.166	6,103.492	2.01%	80.470
15^*^60	PSO	9,091.521	9,950.73	9,456.331	–	91.231
	IA	9,210.278	9,995.259	9,592.015	1.29%	97.778
	GA	9,698.788	11,177.334	10,534.336	6.26%	93.544
	DE	9,917.568	10,815.637	10,327.333	8.43%	92.102

**Table 8:** Scale analysis of the group 3.

**Nodes**	**Algorithm**	**Objective function value**			**GAP**	**CPU time (s)**
		*Z* ^*^	*Z*−*MAX*	*Z*−*AVG*		
20^*^20	PSO	632.999	936.483	759.6515	–	42.126
	IA	849.089	1,173.23	1,015.6294	25.20%	43.546
	GA	820.388	1,235.643	998.1089	23.89%	43.255
	DE	904.581	1,168.351	1,059.019	28.27%	42.459
20^*^30	PSO	1,506.62	1,798.516	1,648.0398	–	56.745
	IA	1,662.516	2,567.568	2,066.8692	20.26%	59.455
	GA	1,882.057	2,798.983	2,500.5531	34.09%	57.642
	DE	1,964.425	2,736.591	2,283.3327	27.82%	56.593
20^*^40	PSO	2,230.89	2,952.151	2,501.9441	–	73.941
	IA	2,569.914	3,290.935	2,950.2068	15.19%	78.366
	GA	2,609.005	3,149.086	2,885.3733	13.29%	72.031
	DE	2,582.686	3,054.551	2,837.5028	11.83%	72.603
20^*^50	PSO	4,602.979	5,212.538	4,964.0735	–	85.426
	IA	5,106.732	5,611.11	5,342.7636	7.09%	89.484
	GA	5,004.926	5,852.391	5,389.0979	7.89%	83.749
	DE	5,255.002	5,966.101	5,576.6224	10.98%	83.519
20^*^60	PSO	8,445.469	10,071.947	8,973.9028	–	98.203
	IA	9,201.623	10,543.646	9,928.9542	9.62%	105.628
	GA	8,616.743	10,223.87	9,201.7031	2.48%	99.946
	DE	8,816.551	10,913.478	9,851.5502	8.91%	98.023

## 7. Conclusions

A robust optimization model was established in this paper to minimize the total expected death probability of casualties in the early stage of emergency rescue after an earthquake, considering the characteristics such as casualty classification, injury deterioration, point-edge mixed failures, medical resource limitation, and casualty number uncertainty. An improved PSO is proposed to solve the problem. Numerical experiments are conducted to verify the effectiveness of the model and algorithm under the context of the Lushan earthquake in China.

The results reveal that the operation effect of the emergency rescue network is significantly improved, and the optimization results are more reliable if the failure scenario is considered in advance. Moreover, robust optimization considering the casualty uncertainty can reduce the uncertainty risk of the system. Therefore, it is necessary to consider the point-edge mixed failures and casualty uncertainty simultaneously in the design stage of the emergency rescue network to establish a more reliable emergency rescue network.

Future research can consider the characteristics of the later stage of post-earthquake rescue comprehensively to study the casualty scheduling problem in the post-earthquake emergency recovery period while considering factors such as resuming normal passage of affected points and transporting the injured.

## Data availability statement

The raw data supporting the conclusions of this article will be made available by the authors, without undue reservation.

## Author contributions

YZ: writing and funding acquisition. YG: modeling and methodology. XH: data, algorithm, and computation. All authors contributed to the article and approved the submitted version.
