# Supplementary material for: Robust optimization for casualty scheduling considering injury deterioration and point-edge mixed failures in early stage of post-earthquake relief
Source: Front Public Health. 2023 Feb 20;11:995829. doi: 10.3389/fpubh.2023.995829 (PMC9986281; doi:10.3389/fpubh.2023.995829)
Supplement: Supplementary file 1 [file Data_Sheet_1.docx]

**Supplementary Material**

**Table S1** The nominal number of casualties at each affected point

| Num. | Affected point | Number of casualties (person) | |
| --- | --- | --- | --- |
|  |  | State *r* | State *g* |
| 1 | Luyang town | 202 | 292 |
| 2 | Feixianguan town | 69 | 124 |
| 3 | Siyan township | 94 | 142 |
| 4 | Qingren township | 145 | 177 |
| 5 | Shuangshi town | 79 | 147 |
| 6 | Longmen township | 223 | 416 |
| 7 | Baosheng township | 58 | 100 |
| 8 | Taiping town | 87 | 122 |
| 9 | Dachuan town | 28 | 40 |

**Table S2** The number of available resources in medical points

| Num. | Candidate medical points | Available resources (units) |
| --- | --- | --- |
| 1 | Lushan middle school | 1000 |
| 2 | Feixian central school | 900 |
| 3 | Siyan Health Center | 900 |
| 4 | Qingren central school | 1000 |
| 5 | Shuangshi middle school | 900 |
| 6 | Zhonglin central school | 800 |
| 7 | Dachuan Health Center | 600 |

**Table S3** The distance between medical points and affected points (km)

| Affected points | Medical points | | | | | | |
| --- | --- | --- | --- | --- | --- | --- | --- |
|  | 1 | 2 | 3 | 4 | 5 | 6 | 7 |
| 1 | 3 | 16 | 7 | 6 | 17 | 42 | 86 |
| 2 | 17 | 3 | 14 | 22 | 35 | 57 | 102 |
| 3 | 6 | 12 | 2 | 13 | 46 | 46 | 92 |
| 4 | 6 | 19 | 12 | 2 | 13 | 42 | 86 |
| 5 | 18 | 33 | 25 | 13 | 2 | 46 | 92 |
| 6 | 17 | 30 | 23 | 17 | 22 | 26 | 70 |
| 7 | 27 | 41 | 34 | 27 | 32 | 14 | 59 |
| 8 | 35 | 49 | 42 | 36 | 45 | 5 | 50 |
| 9 | 82 | 95 | 88 | 81 | 86 | 43 | 3 |

**Table S4** Failure scenarios of medical points

| Medical points | 1 | 2 | 3 | 4 | 5 | 6 | 7 |
| --- | --- | --- | --- | --- | --- | --- | --- |
| scenario 1 | 1 | 1 | 1 | 1 | 1 | 1 | 1 |
| scenario 2 | 1 | 0 | 1 | 1 | 1 | 1 | 1 |
| scenario 3 | 1 | 1 | 1 | 0 | 1 | 1 | 1 |
| scenario 4 | 1 | 1 | 1 | 1 | 1 | 1 | 0 |

**Table S5** Degree of route damage between affected points and medical points

| Affected points | Medical points | | | | | | |
| --- | --- | --- | --- | --- | --- | --- | --- |
|  | 1 | 2 | 3 | 4 | 5 | 6 | 7 |
| 1 | 0.65 | 0.2 | 0.6 | 0.7 | 0.55 | 0.3 | 0.2 |
| 2 | 0.45 | 0.3 | 0.35 | 0.45 | 0.3 | 0.2 | 0.15 |
| 3 | 0.5 | 0.4 | 0.4 | 0.45 | 0.35 | 0.25 | 0.2 |
| 4 | 0.35 | 0.3 | 0.7 | 0.6 | 0.45 | 0.35 | 0.25 |
| 5 | 0.4 | 0.3 | 0.35 | 0.45 | 0.4 | 0.2 | 0.3 |
| 6 | 0.6 | 0.35 | 0.6 | 0.35 | 0.2 | 0.55 | 0.3 |
| 7 | 0.2 | 0.25 | 0.4 | 0.3 | 0.25 | 0.3 | 0.1 |
| 8 | 0.3 | 0 | 0.25 | 0.35 | 0.2 | 0.4 | 0.3 |
| 9 | 0.1 | 0.25 | 0.2 | 0.15 | 0.35 | 0 | 0.2 |

**Table S6** Treatment information at affected points

| Affected points | $k_{ir}$ | $k_{ig}$ | $k_{iw}$ |
| --- | --- | --- | --- |
| 1 | 161 | 292 | 453 |
| 2 | 55 | 124 | 179 |
| 3 | 75 | 142 | 217 |
| 4 | 116 | 177 | 293 |
| 5 | 63 | 147 | 210 |
| 6 | 178 | 416 | 594 |
| 7 | 42 | 100 | 142 |
| 8 | 63 | 122 | 185 |
| 9 | 20 | 40 | 60 |
